# Strategy for Conjugating Oligopeptides to Mesoporous Silica Nanoparticles Using Diazirine-Based Heterobifunctional Linkers

**DOI:** 10.3390/nano12040608

**Published:** 2022-02-11

**Authors:** Md Arif Khan, Ramy W. Ghanim, Maelyn R. Kiser, Mahsa Moradipour, Dennis T. Rogers, John M. Littleton, Luke H. Bradley, Bert C. Lynn, Stephen E. Rankin, Barbara L. Knutson

**Affiliations:** 1 Department of Chemical and Materials Engineering, University of Kentucky, Lexington, KY 40506-0046, USA; makhan226@uky.edu (M.A.K.); Ramy.Ghanim@uky.edu (R.W.G.); maelyn.kiser@uky.edu (M.R.K.); mahsa.moradipour93@gmail.com (M.M.); 2 Naprogenix Inc., UK-ASTeCC, Lexington, KY 40506-0286, USA; dennistrogers@gmail.com (D.T.R.); john.littleton@uky.edu (J.M.L.); 3 Department of Neuroscience, University of Kentucky, Lexington, KY 40536-0298, USA; lhbradley@uky.edu; 4 Department of Chemistry, University of Kentucky, Lexington, KY 40506-0055, USA; bclynn2@uky.edu

**Keywords:** mesoporous silica, nanoparticle, conjugation, oligopeptide, heterobifunctional linker, diazirine

## Abstract

Successful strategies for the attachment of oligopeptides to mesoporous silica with pores large enough to load biomolecules should utilize the high surface area of pores to provide an accessible, protective environment. A two-step oligopeptide functionalization strategy is examined here using diazirine-based heterobifunctional linkers. Mesoporous silica nanoparticles (MSNPs) with average pore diameter of ~8 nm and surface area of ~730 m^2^/g were synthesized and amine-functionalized. Tetrapeptides Gly-Gly-Gly-Gly (GGGG) and Arg-Ser-Ser-Val (RSSV), and a peptide comprised of four copies of RSSV (4RSSV), were covalently attached via their N-terminus to the amine groups on the particle surface by a heterobifunctional linker, sulfo-succinimidyl 6-(4,4′-azipentanamido)hexanoate (sulfo-NHS-LC-diazirine, or SNLD). SNLD consists of an amine-reactive NHS ester group and UV-activable diazirine group, providing precise control over the sequence of attachment steps. Attachment efficiency of RSSV was measured using fluorescein isothiocyanate (FITC)-tagged RSSV (RSSV-FITC). TGA analysis shows similar efficiency (0.29, 0.31 and 0.26 mol peptide/mol amine, respectively) for 4G, RSSV and 4RSSV, suggesting a generalizable method of peptide conjugation. The technique developed here for the conjugation of peptides to MSNPs provides for their attachment in pores and can be translated to selective peptide-based separation and concentration of therapeutics from aqueous process and waste streams.

## 1. Introduction

Synthetic organic functional groups that can mimic the biological specificity of host–guest interactions have been used for analysis, sensing and isolation of different biomolecules, especially in affinity column chromatography [1,2,3,4]. Recent progress in supramolecular chemistry has resulted in tailor-made organic functionalities with high selectivity and specificity toward an array of biomolecules and therapeutic ligands, which can be used for their selective separation [5,6]. Synthetic peptides, in particular, have tremendous molecular recognition and selective binding capabilities and a large volume of peptide libraries with different binding properties has been developed during the last two decades [7,8,9]. Oligopeptide mimics of biological binding sites (of longer proteins) on solid supports are durable, reusable and cost-effective media for affinity separations [7].

Mesoporous silica materials are an ideal support for high affinity functional groups due to their high surface area, tunable pore size and ease of surface modification [10,11,12]. Use of mesoporous silica functionalized with affinity binding sites is widespread in chromatography [13,14].The most common biomolecule linking strategy is to functionalize the silica surface with amine groups and then to covalently link the amine moieties with the N-terminus of peptide/protein molecules. Bifunctional linkers [15,16,17] for amine–amine conjugation can be mainly divided into two types: homo-bifunctional and hetero-bifunctional. Homo-bifunctional linkers such as bis(sulfosuccinimidyl)suberate (BS^3^) have amine-reactive NHS ester groups at both ends. While developed to crosslink two proteins or peptides in solution, they can hypothetically react with amine-functionalized particles at one end and with the amine terminal group of a peptide at the other to covalently attach the peptide to the surface [15,18,19]. During peptide conjugation to amine-functionalized porous particles, competitive rapid hydrolysis of the ester groups of homo-bifunctional linkers poses a significant problem during attachment, and proteins may be physically adsorbed rather than attaching covalently [20,21,22]. Controlling the rate of reaction of the linker with both the peptide and the surface is also challenging. Because of this, homo-bifunctional linkers lack specificity and precise control of attachment orientation.

A hetero-bifunctional linker containing an amine-reactive end and a UV-activable end overcomes the limitations of homo-bifunctional linkers for peptide conjugation. The activation of the UV active group by a light source provides precise control over the attachment mechanism and location. One of the most common hetero-bifunctional linkers for peptide/protein conjugation is the phenyl azide-based sulfosuccinimidyl 6-(4-azido-2-nitrophenyl-amino) hexanoate (sulfo-SANPAH) [20,23,24]. The UV-activable end of sulfo-SANPAH is activated at a wavelength < 320 nm (as low as 260 nm) [15,25], which can cause denaturation of proteins. In addition, the large size of the aromatic photoactive group in sulfo-SANPAH can create steric hindrance [26]. On the other hand, the diazirine group is activated at higher wavelength (340–370 nm) and, thus, does not cause denaturation of proteins and peptides. As a result, diazirine has been used extensively as a photo-labeling agent for proteins [25,27,28]. Diazirine-based photoreactive linkers have better stability compared to commonly used aromatic azide photo-linkers [29,30]. The linkers can also be designed to provide optimal peptide orientation for ligand attachment from the bound surface using spacers. For example, succinimidyl 6-(4,4′-azipentanamido) hexanoate (NHS-LC-diazirine) provides a sufficient spacing arm for the biomolecule to avoid effects of surface interactions on its properties [31]. The NHS-LC-diazirine linker has been shown to be stable under ambient lighting conditions [31,32]. The derivative sulfo-NHS-LC-diazirine (SNLD) contains a charged sulfate group that enhances its aqueous solubility and as a result can be used in physiological media [33].

Covalently linking peptides on the inner surface of the pores of mesoporous silica nanoparticles using hetero-bifunctional linkers has the potential to utilize their high pore volumes, but has limited applicability to mesoporous silica with insufficient pore size (<4 nm) synthesized by traditional templating methods [34,35]. Large pores are required for peptides and linkers to infiltrate the interior surface without pore blockage, as discussed in recent reviews of protein loading in mesoporous silica-based materials [36,37]. Having pores slightly larger than proteins has been shown to allow them to fully access the large pore volume of MSNPs while providing protection from factors such as temperature, solvent, pH and proteolytic hydrolysis [38,39,40]. Immobilizing peptides and proteins inside of mesoporous silica allows their use in diverse applications including therapeutic drug delivery, enzymatic catalysis in harsh environments and for affinity separations [41,42,43]. The hydrodynamic radius of proteins and polypeptides varies considerably depending on folding, but typical sizes are less than 8 nm for denatured proteins with up to 160 residues in their sequence, and considerably more residues for proteins in their native, folded state [44]. Surfactant templating with cationic surfactants such as alkyltrimethylammonium salts typically produces pore sizes of ~3 nm in diameter [45,46,47]. Only recently has surfactant templated synthesis of MSNPs with <170 nm particle diameter and pore sizes of 4.5 nm to 8 nm been reported by using pore expanding agents [48,49]. These particles facilitate the conjugation of long peptide sequences and proteins along with sufficient spacer arms to prevent strong surface effects. A study of peptide conjugation to the inner surface of silica microbubbles (cavity size: 0.55–0.65 mm) using NHS-diazirine has been reported [50]. However, the pore size of a microbubble is thousands of times larger than MSNPs, so there is a need to investigate the use of NHS-diazirine linkers in pores comparable in size to peptides and proteins. Based on previous studies [20,50,51], we hypothesize that effective attachment strategies for oligopeptides within expanded mesopores (>4 nm diameter) can be developed using NHS-diazirine linkers to the peptide N-terminus.

**Figure 1:**
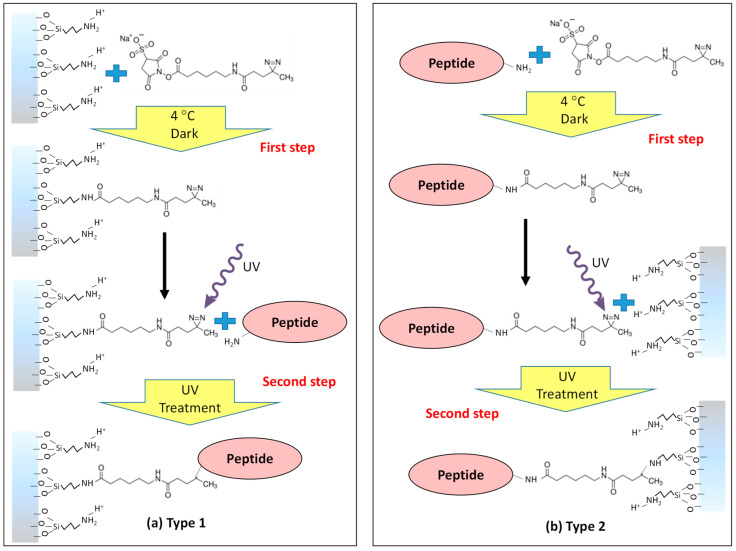
Schematic diagram of the peptide attachment strategies using hetero-bifunctional cross-linker Sulfo-NHS-LC-Diazirine (SNLD): (**a**) Type-1 attachment of the linker to the particle amine group first using the NHS group and then attaching to the peptide amine group using the UV-reactive diazirine group and (**b**) Type-2 attachment of the linker to the peptide amine group first using the NHS group and then attaching to the particle amine group using the UV-reactive diazirine group.

This work examines strategies to conjugate functional oligopeptides to large-pore amine-functionalized MSNPs (~8 nm diameter pores) using the diazirine-based hetero-bifunctional linker sulfo-NHS-LC-diazirine (SNLD) with a spacer of 1.25 nm (SNLD extended length [52]). The model system of four-amino-acid peptide RSSV (Arg-Ser-Ser-Val) was selected for this application from a combinatorial peptide library based on its ability to bind *β*-estradiol (equilibrium constant, *K* = 6 × 10^4^ M^−1^) via column chromatography with good selectivity versus other steroids [53]. Conjugation of the peptide 4RSSV (Arg-Ser-Ser-Val-Arg-Ser-Ser-Val-Arg-Ser-Ser-Val-Arg-Ser-Ser-Val), a four-repeat peptide of the original RSSV 4-mer, and 4G (Gly-Gly-Gly-Gly) to the nanoparticles is investigated to test the versatility of the conjugation strategy for peptides of varying length and amino acid sequence. Two conjugation strategies are proposed based on the sequence of attaching the linker to the particle or peptide (Figure 1). For Type-1 attachment, the linker is first attached to the surface amine group using the NHS-ester and then the peptide amine group is attached to the linker using UV activation of diazirine. For Type-2 attachment, the linker is first attached to the peptide using the NHS-ester followed by attachment to the surface using UV activation. The anticipated advantage of Type-1 attachment is that unbound linkers can be removed by washing after the first step and before attachment to peptides. However, activated diazirine is indiscriminate toward N-H or O-H moieties and can result in attachment to either terminus of the peptide. Activation of the diazirine group under UV light has also been shown to bind the carboxyl moieties of proteins to some degree [54]. Another potential problem with Type-1 attachment in a porous system is the possible attachment of the diazirine moiety with another amine group inside the pores. Specific attachment of the peptide N-terminus to the linker during the first step is the main advantage of Type-2 attachment. In addition, the peptide-attached linker can diffuse into the pore prior to the photoactivated conjugation to the surface, promoting reactions within the pores and not just at the surface. Attachment of diazirine to O-H moieties of particles provide no disadvantages (in fact, it is probably advantageous in keeping some of amine groups unattached and positively charged) compared to Type-1 attachment. However, during the second step of Type-2 attachment, the activated diazirine of the peptide-attached linker can react with the C-terminus of another peptide. These solution-based complexes would be removed during washing but would result in inefficient use of peptide and linker. Considering these pros and cons, the attachment efficiency is examined for both of these methods using fluorescence spectroscopy of fluorescein isothiocyanate (FITC)-labeled peptide and thermogravimetric (TGA) analysis.

## 2. Materials and Methods

### 2.1. Chemicals and Reagents

Tetrapropyl orthosilicate (TPOS, 95%), phosphate-buffered saline (PBS) tablets, (3-aminopropyl)triethoxysilane (APTES, 99%) and N,N-dimethylformamide (DMF, molecular biology grade) were purchased from Sigma-Aldrich (St. Louis, MO, USA). Cetyltrimethylammonium bromide (CTAB, 99.8%) was purchased from MP Biomedicals (Solon, OH, USA); NaOH pellets (≥97%) from EMD Millipore; acetone (≥99.5%) from BDH analytical; 1,3,5-triisopropylbenzene (TIPB, >95%), triethanolamine (TEA, >98%) and fluorescamine from Alfa-Aesar (Tewksbury, MA, USA); and ultrapure deionized ultra-filtrated (DIUF) water, ethanol (200 proof) and 12 N HCl (ACS grade) from Fisher Scientific (Pittsburgh, PA, USA). Heterobifunctional linker sulfo-NHS-LC-diazirine (SNLD, Pierce^TM^) was purchased from Thermo Fisher Scientific (Pittsburgh, PA, USA) and used for peptide conjugation.

*Oligopeptide synthesis*. Peptide 4G (GGGG) was obtained from Sigma-Aldrich (St. Louis, MO, USA); RSSV and 4RSSV were synthesized by Genscript (Piscataway, NJ, USA); and RSSV-FITC was synthesized by Lifetein (Hillsborough, NJ, USA) using solid-phase peptide synthesis and purified to >95% yield using reverse phase high-pressure liquid chromatography (RP-HPLC). RSSV-FITC was designed (sequence Arg-Ser-Lys(FITC)-Val) by replacing the serine at position 3 with a lysine to incorporate the fluorescein isothiocyanate (FITC) fluorescent label while maintaining the peptide arginine positive charge for peptide function and the single free amine (N-terminus) for attachment. All peptide sequences were confirmed to be of the correct molecular mass by LC-MSMS analysis following purification. Lyophilized peptide was resuspended in PBS buffer (pH 7.4) to a final concentration of 1.7 mg/mL 4G, 3 mg/mL RSSV or 12 mg/mL 4RSSV before conjugation to particles.

### 2.2. Mesoporous Silica Nanoparticles (MSNPs) Synthesis

MSNPs were synthesized following the method described by Yamada et al. [49], where TIPB was used to swell the CTAB micelles, the pore forming agent, during surfactant-templated synthesis. Initially, 0.56 mL of TEA and 3.0 g of CTAB were added to 360 mL of DIUF water. The solution was stirred at 80 °C for 2 h for complete mixing and emulsion formation, and 16 mL of TIPB was added under vigorous mixing. After 30 min, a complete colloidal state (oil-in-water) was obtained and 4.77 mL of TPOS was added with constant stirring. The solution was stirred vigorously for 12 h at 80 °C to obtain white solid particles. The particles were then separated by repeated centrifugation and washing, and the surfactant was removed by acidic ethanol (2 M HCl in ethanol) washing before drying at 84 °C overnight.

### 2.3. Amine Functionalization and Quantification

Amine-functionalized MSNPs (MSNPAs) were obtained by condensing APTES on the particle surface using modified literature procedures [10,55,56,57]. Two-hundred milligrams of MSNPs were uniformly dispersed in 25 mL of dry ethanol by sonication for 15 min. An amount of 0.5 mL of APTES was added dropwise under constant stirring in a nitrogen-filled glove bag, and the solution was kept stirring in a closed vessel for 24 h at room temperature. Particles were centrifuged with repeated ethanol washing and cured at 84 °C for 24 h. After curing, particles were stirred in excess ethanol for 24 h to remove any remaining loosely bound amine species. The functionalized particles were again washed 3 times with anhydrous ethanol and dried at 84 °C.

*Amine quantification.* The amount of amine groups on the functionalized particle surface was determined by a previously reported fluorescamine assay after particle dissolution [58,59]. A total of 30 mg of particles was dissolved over an 8 h period in 30 mL of 0.02 M NaOH at room temperature under vigorous stirring. One-hundred microliters of this solution and 1.0 mL of 1.0 mM fluorescamine in acetone were mixed with 2.0 mL of PBS solution at pH 7.4. The emitted fluorescence intensity of this solution was measured at 480 nm after excitation at 366 nm using an Agilent (Santa Clara, CA, USA) Varian Cary Eclipse fluorescent spectrophotometer. The amount of amine on the particle surface was determined by a calibration curve prepared using known amounts of APTES and non-functionalized MSNPs.

### 2.4. Peptide Attachment to MSNPAs

For Type-1 attachment, 10 mg of MSNPAs were sonicated in 1 mL of PBS solution (pH 7.4) for 15 min to make a uniform dispersion and mixed with 3 mg of SNLD in 100 µL DMF at 4 °C. The mixture was stirred at 4 °C overnight followed by centrifugation of the particles. Particles were washed with fresh PBS solution three times to remove excess, unbound linker and then dispersed in 2 mL of PBS solution containing 3 mg of RSSV peptide at room temperature with vortex mixing. The solution was then stirred overnight to allow adsorption of peptide on the particle surface and finally treated with UV light (Thorlabs, Newton, NJ, USA, model M00284926, λ = 365 nm, 1.2 A) with continuous stirring for 60 min, which was selected based on a series of attachment experiments using RSSV-FITC with different UV treatment times (0, 10, 30, 45, 60, 90 and 120 min) (see Section 2.6 for quantification). After UV treatment, peptide-conjugated particles (MSNPA-RSSV) were separated by centrifugation and washed 5 times with fresh PBS solution and dried overnight in vacuum at room temperature.

For the Type-2 attachment, 3 mg of SNLD in 100 µL DMF was mixed with 3 mg of RSSV peptide in 1 mL PBS solution, and the mixture was allowed to stir at 4 °C overnight for the completion of linking with the amine terminus of peptide. Ten milligrams of MSNPAs was dispersed uniformly in 1 mL PBS with sonication and added to the peptide-linker solution. The combined mixture was allowed to stir overnight for adsorption of peptide–linker conjugation on the particle surface and then UV treated for 60 min with continuous stirring to allow for the attachment to the particle surface. Finally, peptide-attached particles were separated by centrifugation, washed thoroughly with fresh PBS solution and dried overnight in vacuum at room temperature. 4G and 4RSSV attachment using the Type-2 attachment method is similar, but 1.7 mg of 4G or 12 mg of 4RSSV was used to keep the molar ratio of peptide:amine approximately the same as for RSSV.

### 2.5. Material Characterization

A FEI (Hillsboro, OR, USA) Helios Nanolab 660 Focused Ion Beam/Scanning Electron Microscope (SEM) was used to examine the particle morphology. Particles were dispersed onto a 15 mm aluminum stub using double-sided carbon tape, excess materials were blown off with dry N_2_ and the samples were stored in a desiccator for 24 h. Prior to SEM analysis, the particles were coated with conductive Au-Pd alloy using an Emscope (Hercules, CA, USA) SC400 sputtering system. Average and standard deviation of particle diameters were calculated using 20 random particles using ImageJ Software, version 1.53. The internal pore structure of the particles was characterized using a FEI (Hillsboro, OR, USA) Talos F200X Transmission Electron Microscopy (TEM). Particles were dispersed in ethanol within a sonication bath for 5 min. Using a pipette, a small volume of the suspension was drop cast onto a lacey carbon-coated copper TEM grid, which was allowed to dry for a minimum of 5 min. Afterward, the samples were stored in a TEM storage box overnight before imaging. Surface characterization was performed using nitrogen adsorption conducted at −196 °C with a Micromeritics (Norcross, GA, USA) TriStar 3000 gas sorption instrument. Samples were degassed at 135 °C for 4 h under flowing N_2_ gas before analysis. The specific surface area, average pore diameter and pore size distribution were estimated using the Brunauer–Emmett–Teller (BET) isotherm and by Barrett–Joyner–Halenda (BJH) method, respectively. To confirm the covalent linkage of peptides, Fourier transform infrared (FTIR) spectroscopy was conducted using a Thermo Fisher Scientific (Pittsburgh, PA) Nicolet Nexus 470 spectrometer with a deuterated triglycine sulfate (DTGS) detector. A total of 0.5 g of anhydrous KBr and particles (0.5–1.0 wt%) was crushed with a mortar and pestle, and some of this powder was pressed into a pellet for transmission analysis. Dynamic light scattering (DLS) was used to measure the zeta potential of the particles with an Anton-Paar (Ashland, VA, USA) Lightsizer 500 instrument. Initially, a 1 mg/mL uniform particle suspensions was made in DIUF water with sonication and diluted to around 0.1 mg/mL concentration before measurement. The pH values of the solutions were adjusted by adding a very small amount of either 0.1 N HCl or 0.1 N NaOH solution in water as required to obtain the desired pH, which was checked before every measurement with a benchtop pH meter (Accumet Research AR25 dual channel pH meter from Fisher Scientific, Pittsburgh, PA, USA). For measurements, the solutions were carefully placed in an Omega Cuvette consisting of an inverted omega-shaped capillary tube without any air bubbles.

### 2.6. Quantification of Peptide Attachment

Fluorescein isothiocyanate (FITC)-labeled RSSV peptide (RSSV-FITC) was used to quantify RSSV attachment efficiency to the particle amine groups by solution depletion with fluorescence spectroscopy. During quantification of peptide attachment, the amount of particles and linkers and the solution volume were the same as during RSSV attachment. The amount of RSSV-FITC was adjusted to 5.5 mg (instead of 3 mg RSSV) to keep the molar ratio of peptide:amine the same. Fluorescence intensity of the solution after attachment was measured at an emission wavelength of 520 nm (peak fluorescence) after excitation at 495 nm (peak absorbance) and compared to a calibration curve prepared with known amounts of RSSV-FITC. Type-1 attachment was quantified using the calibration curve of RSSV-FITC (only peptide) fluorescence intensity, whereas Type-2 attachment was quantified using the calibration curve of SNLD-RSSV-FITC (peptide conjugated with linker) after correcting to account for photo-bleaching (intensity reduction) during the UV treatment period.

Thermogravimetric analysis (TGA) was performed to quantify the amount of organic groups (peptides) conjugated to the particle surface with a TA-SDT-Q600 simultaneous TGA/DSC instrument (TA Instruments, New Castle, DE, USA). Particle samples were dried at 50 °C under vacuum overnight before performing TGA analysis from 25 °C to 500 °C with a ramp rate of 10 °C/min and under constant dry air flow of 100 mL/min. Functional group contents were analyzed by thermal decomposition and combustion of organics for MSNPA, MSNPA-4G, MSNPA-RSSV and MSNPA-4RSSV compared to bare MSNP. Specifically, mass losses from 150 °C to 500 °C were used to determine the amount of amine grafting (before conjugation) and peptide grafting (after conjugation).

## 3. Results and Discussion

**Figure 2:**
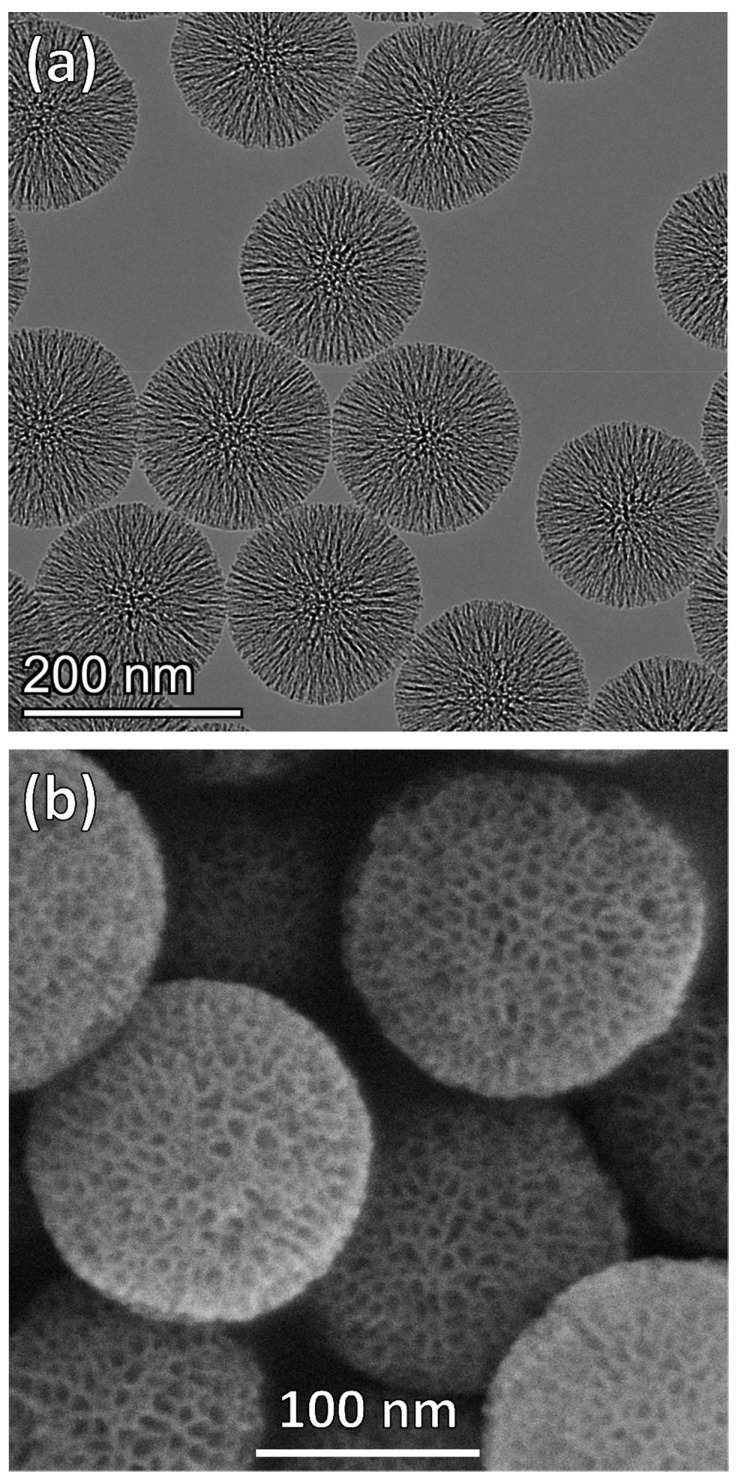
Representative (**a**) transmission electron micrograph and (**b**) scanning electron micrograph of bare MSNPs showing spherical particles with average particle diameter 146 ± 27 nm and radially oriented mesopores with an average pore diameter of 8 nm.

MSNPs with large pores (~8 nm average pore size) were synthesized by the method of Yamada et al. [49], where CTAB surfactant template was used to create pores and TIPB served to expand the micelle pore templates. The conditions used here were intended to reproduce the particles reported by Yamada et al. with a TIPB:CTAB molar ratio of 8, which were shown to have an open, accessible pore morphology. After synthesis, the surfactants were removed from the pores by acidic washing. Particles were then functionalized with amine groups by the condensation of the organosilane precursor, APTES, on the particle and pore surface. Spherical particles with average diameter of 146 ± 27 nm and radially oriented, 8 nm pores were obtained after template extraction, as seen in the TEM and SEM image presented in Figure 2. This particle size is consistent with the size reported by Yamada et al. [49] and suitable for cell uptake [60].

Surface characterization (surface area, pore volume and average pore size) was performed using nitrogen adsorption before and after amine functionalization (Figure S1 in electronic supplementary information (ESI) and Table 1). Nitrogen sorption of particles showed Type-IV isotherms (Figure S1a), consistent with the presence of uniformly sized mesopores and textural porosity giving an uptick in adsorption at high relative pressure. X-ray diffraction patterns did not show any peaks (data not shown), as the radially oriented pores (Figure 2a) do not have large domains of uniformly oriented mesopores. The average pore diameter, as determined by the BJH method, was reduced with functionalization (7.9 nm to 7.6 nm) (Figure S1b). The pore size distribution of the MSNP base material matches that reported by Yamada et al. for particles prepared with 8:1 TIPB:CTAB ratio [49]. The width of the pore size distribution is consistent with the radial mesopore structure evident in Figure 2b. The surface area and pore volume were also reduced after functionalization, consistent with amine grafting inside the mesopores. The large pore size of the particles (relative to CTAB-templating alone) allows the amine functional groups in the pores to be accessible for covalent attachment to the linkers and peptides. The amount of amines on the particle surface was estimated by chemical analysis to be 1.53 mmol amine/g silica, corresponding to 64% of a monolayer coverage on the particle surface considering the projected area per aminopropyl group on the surface (0.5 nm^2^/aminopropyl group) [58,61].

Oligopeptides RSSV, 4G and 4RSSV were conjugated to amine functional groups on the particle using hetero-bifunctional linker SNLD, which provides a combination of amine-reactive chemistry with the photochemistry of diazirine groups for UV activation. The silica pore walls should not hinder UV light reaching the interior of the particles; silica cavities with pore walls of much greater thickness (2 µm) have been functionalized using a similar UV treatment process [50]. UV-activated conjugation is relatively rapid and efficient compared to NHS conjugation [62] and was determined to be optimized with 60 min of treatment using RSSV-FITC attachment (data not shown).

For RSSV, two different peptide conjugation strategies were used with a bifunctional linker (Figure 1). For Type-1 attachment, SNLD was first conjugated to amine groups on the particles by using amine-reactive NHS moieties, and then the peptide N-terminus was attached to the linker using the diazirine end via UV treatment. In the Type-2 attachment sequence, the peptide N-terminus was first attached to the linker NHS end before attaching the diazirine end to particle amine groups by UV treatment. FITC-conjugated RSSV (RSSV-FITC) was used to evaluate these two oligiopeptide attachment strategies. Fluorophore-tagged peptides/proteins are widely used to calculate the attachment efficiency using homo- or hetero-bifunctional linkers [63]. The UV–Vis absorbance spectra of RSSV-FITC were measured (Figure S2 of ESI), where the absorbance peak and intensities do not change with UV treatment up to 120 min of treatment. Fluorescence intensities were used to measure RSSV-FITC attachment by solution depletion, while accounting for photobleaching of the fluorescent moiety (using a control UV treatment in the absence of any particles) during the functionalization process. UV illumination caused 25% and 35% reduction in intensity for RSSV-FITC and SNLD-RSSV-FITC after 60 min of treatment for Type-1 (Figures S3 and S4) and Type-2 attachment (Figures S5 and S6), respectively.

**Figure 3:**
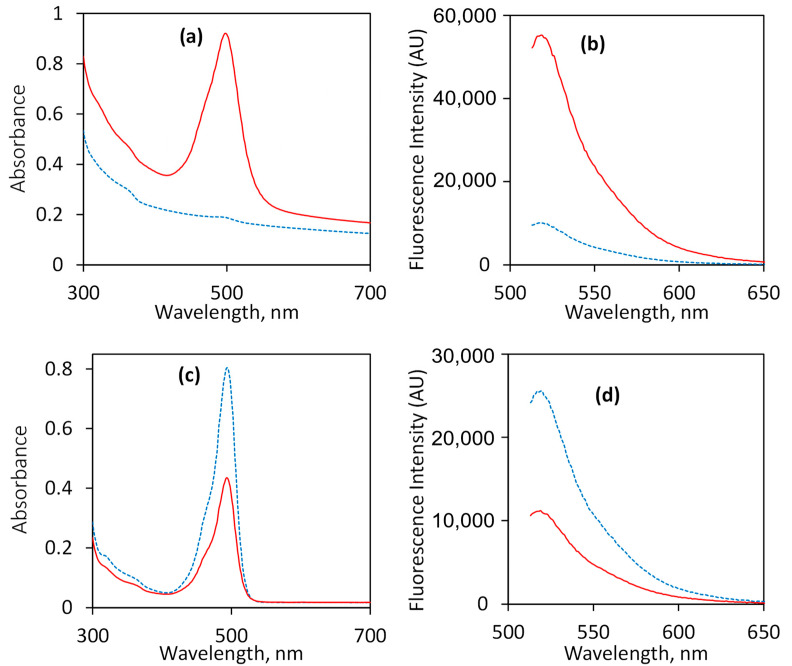
UV–Vis absorbance (left) and fluorescence intensity (right) of (**a**,**b**) particles re-suspended in solution and (**c**,**d**) supernatant after fluorescein isothiocyanate (FITC)-labeled RSSV (Arg-Ser-Ser-Val tetrapeptide) attachment to the particles using Type-2 conjugation. Solid red lines and dashed blue lines represent results with or without UV treatment, respectively. Both UV absorbance and fluorescence intensity of the particles increases after UV treatment, whereas supernatant intensity decreases, suggesting successful binding of peptide.

Based on the calculation of solution depletion after peptide conjugation, the attachment efficiency of Type-2 conjugation was found to be 0.43 mol peptide/mol amine, whereas for Type-1 conjugation, it was 0.24 mol peptide/mol amine. Better efficiency of Type-2 attachment is consistent with literature describing antibody conjugation to polyamine yarns using NHS-LC-diazirine [64]. The improved attachment efficiency may be due to the high concentration of diazirine groups accessible to amine groups in the pores during Type-2 attachment. It is also possible for the diazirine group to bind with another amine group or unintended peptide moieties during Type-1 attachment, which along with lower attachment efficiency, makes Type-1 a less attractive option. During Type-2 attachment, activated diazirine can attach to the carboxyl moiety of another peptide. However, if activated diazirine binds to the C-terminus of another peptide, the complex will be removed during particle washing steps. Thus, Type-2 attachment is preferable to preserve peptide functionality. UV–Vis absorbance and fluorescence intensity of the particles and the supernatant after Type-2 conjugation using RSSV-FITC with UV treatment are provided in Figure 3, compared to identical systems in the absence of UV treatment (no diazirine activation and subsequent covalent bond formation). The absorbance and fluorescence intensity of the particles increased only when they underwent UV treatment, whereas absorbance and fluorescence intensity decreased in the supernatant, consistent with RSSV-FITC attachment to the particles. Note that the particle external surface represents less than 3% of the total surface area (considering spherical particles), and as a result, peptide attachment should be only 0.03 mol peptide/mol amine if peptides were only able to attach to the outer surface amines of the particles. Considering the high relative amount of peptide attachment, the peptides are conjugated primarily to the amines inside the pores, as hypothesized.

**Figure 4:**
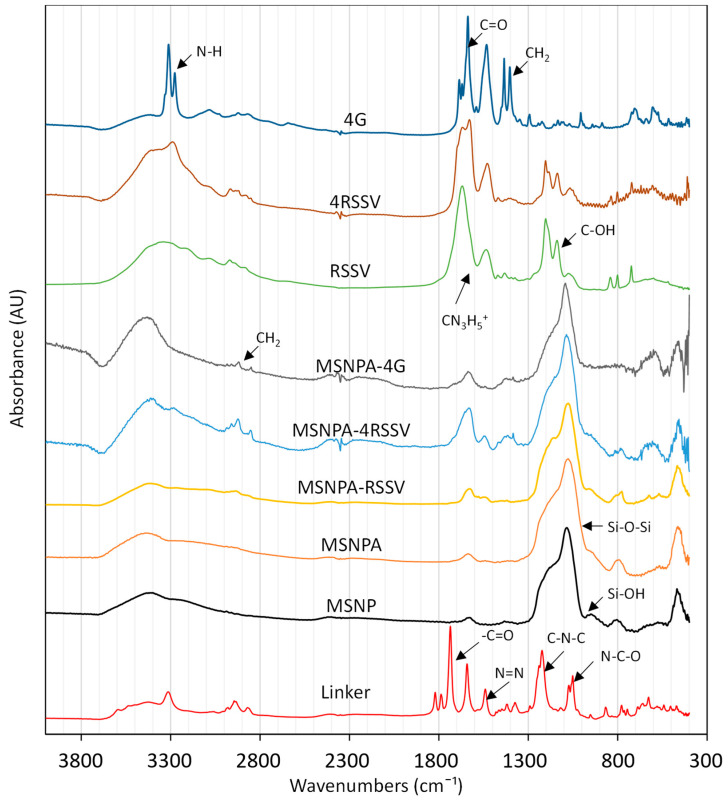
FTIR spectra of peptide functionalized particles relative to bare MSNPs and MSNPAs, as well as fresh linker (sulfo-NHS-LC-diazirine) and peptides with peaks corresponding to major functional groups labeled. In the figure, 4RSSV is a peptide made of 4 sequential RSSV units and 4G is a tetraglycine.

To show covalent linkage (not merely physical adsorption), FTIR spectra of the particles after peptide attachment were measured. The FTIR spectra of MSNPs (bare, amine-functionalized, RSSV-functionalized and 4RSSV-functionalized prepared by Type-2 attachment) are compared in Figure 4, along with the spectra of fresh linker and peptides. The FTIR spectra of the bare MPSNs do not contain a peak due to –CH_2_ stretching (2800–3000 cm^−1^), suggesting complete removal of the organic template following particle synthesis. For bare MSNPs and MSNPAs, the most prominent peaks are bands corresponding to Si-O-Si and Si-OH vibration, located at 1080 and 960 cm^−1^, respectively [38]. Primary amine peaks are not visible, but amine functionalization was quantified by chemical analysis, as described previously. The linker, SNLD, has symmetric and asymmetric (-C=O from the ester) stretching vibrations at 1788 and 1736 cm^−1^, respectively, whereas peaks at 1223 and 1051 cm^−1^ can be assigned as asymmetric C-N-C and N-C-O stretching vibrations, respectively [21]. There are also two peaks correspond to diazirine (N=N stretching at 1643 and N-H amide bond stretching at 1540 cm^−1^) [65,66,67,68]. After peptide attachment, the intensity of –CH_2_ stretching vibrations (2800–3000 cm^−1^) increases, which indicates the presence of linking spacer between particle surface and peptide, whereas peaks corresponding to NHS ester and diazirine groups disappear, suggesting their conversion during the attachment process. Both MSNPA-RSSV and MSNPA-4RSSV show increased intensity corresponding to the arginine side chain stretching vibration, which confirms peptide attachment to the particle surface. Specifically, the spectra of the RSSV oligopeptides have characteristic peaks from the arginine side chain (CN_3_H_5_^+^) (asymmetric and symmetric stretching vibrations of 1673 and 1586 cm^−1^, respectively), -CH_3_ bending vibration at 1460 cm^−1^ from valine and C-OH bending vibration from serine side chain (1181 cm^−1^) [69]. On the other hand, characteristic peaks of the inner groups from 4G (CH_2_ bending at 1435 cm^−1^, COO- symmetric and asymmetric stretching at 1788 and 1736 cm^−1^, and C=O stretching at 1637 cm^−1^ [70]) remain, while N-H symmetric and asymmetric stretching peaks (at 3311 and 3276 cm^−1^ [71]) disappear, suggesting covalent linkage through the N-terminus.

**Figure 5:**
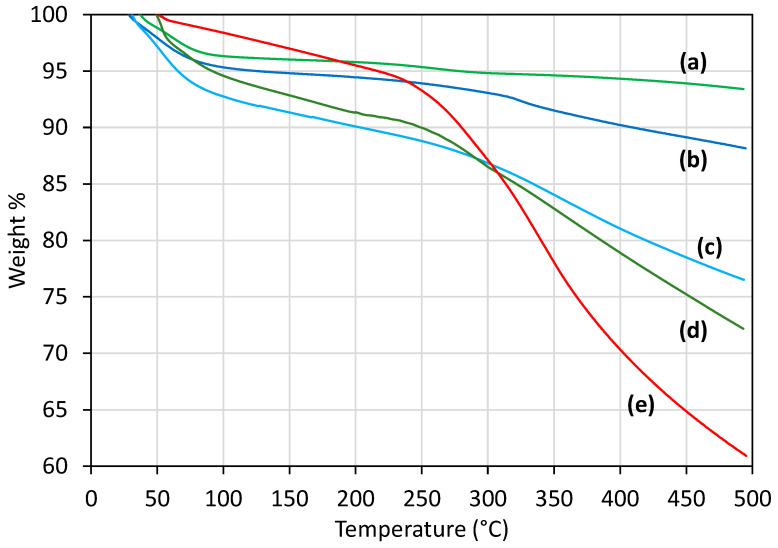
Thermogravimetric analysis (TGA) profiles of particles showing relative mass loss with temperature increase for (**a**) MSNP, (**b**) MSNPA, (**c**) MSNPA-4G, (**d**) MSNPA-RSSV and (**e**) MSNPA-4RSSV.

The quantity of non-fluorescent peptides (RSSV, 4RSSV and 4G) conjugated to the MSPAs was measured directly by TGA to demonstrate that the Type-2 attachment strategy is generalizable. Specifically, the mass loss by the peptide (RSSV, 4G and 4RSSV)–linker-conjugated MSNPAs (synthesized using Type-2 attachment) was compared to that of MSNPs and MSNPAs in the range 150 °C to 500 °C, which corresponds to the thermal degradation and combustion of the organic groups (Figure 5). For bare MSNPs, 2.6% mass is lost from 150 to 500 °C (0.0278 mg/mg silica; representing impurities such as residual template and further temperature-induced condensation of silica) and is subtracted before calculating the organic content of the other particles based on mass loss in this temperature range.

The amount of aminopropyl groups on MSNPAs was found to be 0.494 mg/mg silica (1.04 mmol amine/g silica). Therefore, TGA analysis underestimates the amount of amine by a factor of 0.67 compared to that of chemical analysis (see above). The reason for this underestimation may be the presence of carbon residue on the particle surface (which was visually observed on the particles). After subtraction of the weight loss of organics for MSNPAs, an additional 0.132, 0.208 and 0.516 mg/(mg silica) weight loss (due to removal of peptides and linkers) was observed for MSNPA-4G, MSNPA-RSSV and MSNPA-4RSSV, respectively. This corresponds to 0.300 mmol 4G/g silica, 0.324 mmol RSSV/g silica and 0.267 mmol 4RSSV/g silica. The molar attachment efficiency with amine is similar for the three peptides: 0.288, 0.311 and 0.257 mol peptide/mol amine for 4G, RSSV and 4RSSV, respectively, based on the masses of amines calculated using TGA. It is usually difficult to estimate protein length due to the presence of different secondary structures, but for oligopeptides, an average length of 3.6 Å can be assumed per amino acid [72]. Thus, a contour length of 1.4 nm, 1.4 nm and 5.8 nm can be approximated for 4G, RSSV and 4RSSV, respectively, in addition to 1.25 nm of spacer length from the linker [52]. Therefore, the method of peptide attachment to MSNPAs is robust to amino acid sequences and oligopeptide length. Furthermore, note that TGA analysis underestimated RSSV attachment by a factor of 0.72 compared to fluorescence spectroscopy measurements, which is close to aminopropyl underestimation for MSNPAs, again due to carbon residue on the particle surface. Thus, chemical methods are more accurate in estimation of peptide attachment, but TGA can provide a reasonable estimation of relative attachment.

All of the particles after peptide conjugation (MSNPA-4G, MSNPA-RSSV and MSNPA-4RSSV) remain positively charged at physiological pH (Figure S7), consistent with the presence of unreacted surface amine groups. MSNPA-4G has a slightly lower zeta potential compared to bare MSNPAs, but MSNPA-RSSV and MSNPA-4RSSV have comparable charge to MSNPAs. Possible charge reduction after reaction of surface amine groups due to peptide conjugation may be offset by the charge of the positive moieties of arginine side chains for MSNPA-RSSV and MSNPA-4RSSV. Positive charge is important for the colloidal stability of the particles during suspension and re-suspension. Due to high positive charge, these particles should be appropriate for intracellular penetration where positive charge has been shown to be beneficial [73].

A challenge of covalent immobilization of peptides on surfaces is the possible loss of the desired selective ligand binding functionality after attachment [7]. Selective binding of biomolecules with peptide immobilized to an affinity column surface was demonstrated [7,53], and even utilized for the fractionation of some biomolecules [74,75,76]. For biomolecule binding with covalently linked peptides on particle surfaces, although hydrophobic interactions are the main driving force for binding, hydrogen bonding with hydroxyl and aminopropyl groups may also be present [77,78], which makes the binding more complicated. A large amount of non-specific binding of biomolecules from aqueous solutions is also expected. Here, controlled covalent linkage of peptide to mesoporous silica nanoparticles provides a better opportunity to effectively use the pores, in contrast to noncovalent functionalization [34,79], which has been used for RNA delivery or theranostics. Functionalization inside the pores is also superior compared to only external surface functionalization by peptide for drug delivery [10] or cellular receptor binding [80]. High *β*-estradiol binding capacity of tetrapeptide RSSV [53] and its repeat 4-mer should also provide an opportunity for the removal of these types of compounds from polluted water sources, which are well-recognized endocrine disrupting compounds [81]. However, selective biomolecule binding and separation is beyond the scope of current investigation and will be pursued in future studies.

## 4. Conclusions

Functional oligopeptides 4G (Gly-Gly-Gly-Gly), RSSV (Arg-Ser-Ser-Val) and 4RSSV were attached to large pore (7.9 nm diameter), amine-functionalized MSNPS using a hetero-bifunctional linker, sulfo-NHS-LC-diazirine (SNLD), which contains an amine-reactive NHS ester group and UV-activable diazirine group. Hetero-bifunctional peptide linkers containing a diazirine group provide precise control of the mechanism and orientation during attachment with a high activation wavelength (365 nm), which is more benign to proteins and peptides compared to other linkers that are activated at lower wavelength. Two different conjugation schemes were compared to attach oligopeptide RSSV to the MSNPs based on the order of addition (Type-1: functionalize particle with linker and then attach the peptide; Type-2: attach the linker to the peptide and then functionalize the particle with peptide–linker conjugate). The efficiency of peptide attachment was measured by fluorescence spectroscopy using FITC-labeled peptides (RSSV-FITC). Higher attachment efficiency per mol amine groups was found for Type-2 attachment (0.43 mol RSSV/mol amine) compared with Type-1 attachment (0.24 mol RSSV/mol amine). Type-2 attachment efficiencies of 4G, RSSV and 4RSSV on particles, as determined by TGA analysis, were similar. This demonstrates that the attachment strategy is generalizable and can be used to attach a range of sizes of oligopeptide to MSNPA.

The functional oligopeptide conjugation to “large pore” MSNPs appropriate for biomolecule loading was demonstrated using a versatile and robust hetero-bifunctional linking strategy, which provides precise control of binding moieties of peptide molecules. High-capacity platforms for selective separation of biomolecules with therapeutic value can be designed by selecting oligopeptides that mimic the specific binding sites of biomolecules. For example, MSNPAs can be used to selectively isolate different small molecular therapeutics from living plants, a technique recently demonstrated by using engineered silica nanoparticles [73]. Further, high-capacity adsorbents for the removal of specific therapeutics in polluted water sources can be developed based on functionalizing silica particles with selective members of peptide libraries.

## Figures and Tables

**Table 1:** Surface properties of MSNPs from nitrogen adsorption before and after amine functionalization.

Particle Type	BET Surface Area (m^2^/g)	Total Pore Volume (cm^3^/g)	Average Pore Diameter (nm) ^a^
MSNP	729	2.32	7.9 ± 2.2
MSNPA	469	1.50	7.6 ± 1.9

^a^ The range is determined from the full width at half maximum (FWHM) of the BJH pore size distribution.

## Data Availability

The data presented in this study are available on request from the corresponding authors.

## Supplementary Materials

The following supporting information can be downloaded at: https://www.mdpi.com/article/10.3390/nano12040608/s1, Figure S1: (a) Nitrogen sorption isotherms and (b) pore size distributions of non-functionalized and amine functionalized MSNPs; Figure S2: Absorbance spectra of RSSV-FITC before and after UV exposure; Figure S3: Calibration curve for the concentration of RSSV-FITC in solution; Figure S4: Fluorescence intensity of RSSV-FITC solution during UV photobleaching; Figure S5: Calibration curve for fluorescence intensities of SNLD-RSSV-FITC before UV treatment; Figure S6: Fluorescence intensity of SNLD-RSSV-FITC solution during UV photo-bleaching; and Figure S7: Zeta potentials vs pH for MSNPA-RSSV, MSNPA-4RSSV, MSNPA-4G, and MSNPA.
