# Supplementary material for: Strategy for Conjugating Oligopeptides to Mesoporous Silica Nanoparticles Using Diazirine-Based Heterobifunctional Linkers
Source: Nanomaterials (Basel). 2022 Feb 11;12(4):608. doi: 10.3390/nano12040608 (PMC8880541; doi:10.3390/nano12040608)
Supplement: Supplementary file 1 [file nanomaterials-12-00608-s001.zip › nanomaterials-1475963-supplementary.pdf]

Supplementary Material

Strategy for Conjugating Oligopeptides to Mesoporous Silica Nanoparticles Using Diazirine-Based Heterobifunctional Linkers

Md Arif Khan [1], Ramy W. Ghanim [1], Maelyn R. Kiser [1], Mahsa Moradipour [1], Dennis T. Rogers [2], John M. Littleton [2], Luke H. Bradley [3], Bert C. Lynn [4], Stephen E. Rankin [1,*] and Barbara L. Knutson [1,*]

1 Department of Chemical and Materials Engineering, University of Kentucky, Lexington, KY 40506-0046, USA; makhan226@uky.edu (M.A.K.); Ramy.Ghanim@uky.edu (R.W.G.); maelyn.kiser@uky.edu (M.R.K.); mahsa.moradipour93@gmail.com (M.M.)
2 Naprogenix Inc., UK-ASTeCC, Lexington, KY 40506-0286, USA; dennistrogers@gmail.com (D.T.R.); john.littleton@uky.edu (J.M.L.)
3 Department of Neuroscience, University of Kentucky, Lexington, KY 40536-0298, USA; lhbradley@uky.edu
4 Department of Chemistry, University of Kentucky, Lexington, KY 40506-0055, USA; bclynn2@uky.edu
* Correspondence: stephen.rankin@uky.edu (S.E.R.); bknut2@uky.edu (B.L.K.); Tel: +1-859-257-9799 (S.E.R.); +1-859-257-5715 (B.L.K.)

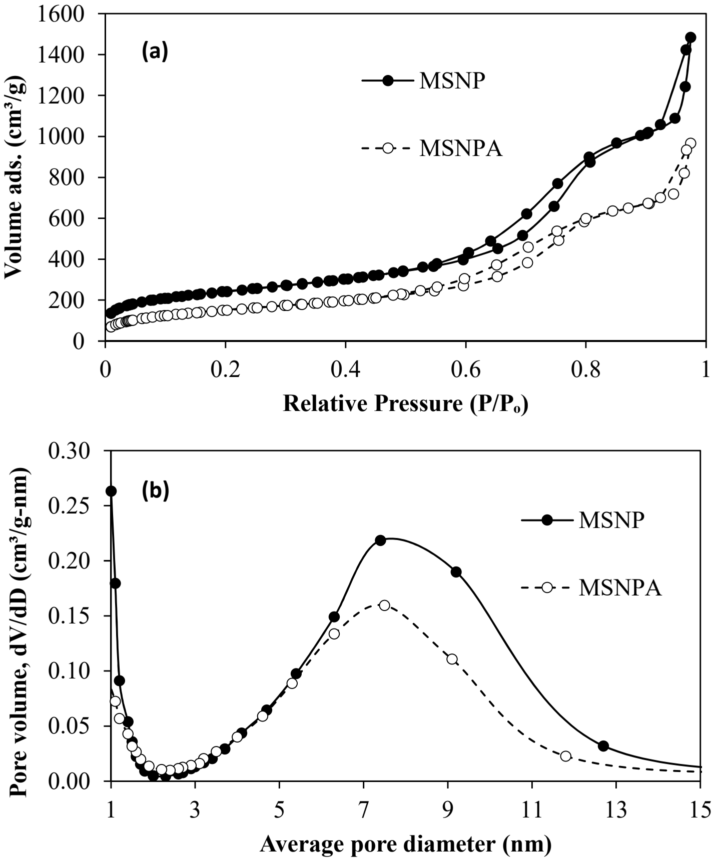

Figure S1. Surface property characterization of non-functionalized and amine functionalized MSNPs: (**a**) nitrogen sorption isotherms and (**b**) BJH pore size distribution.

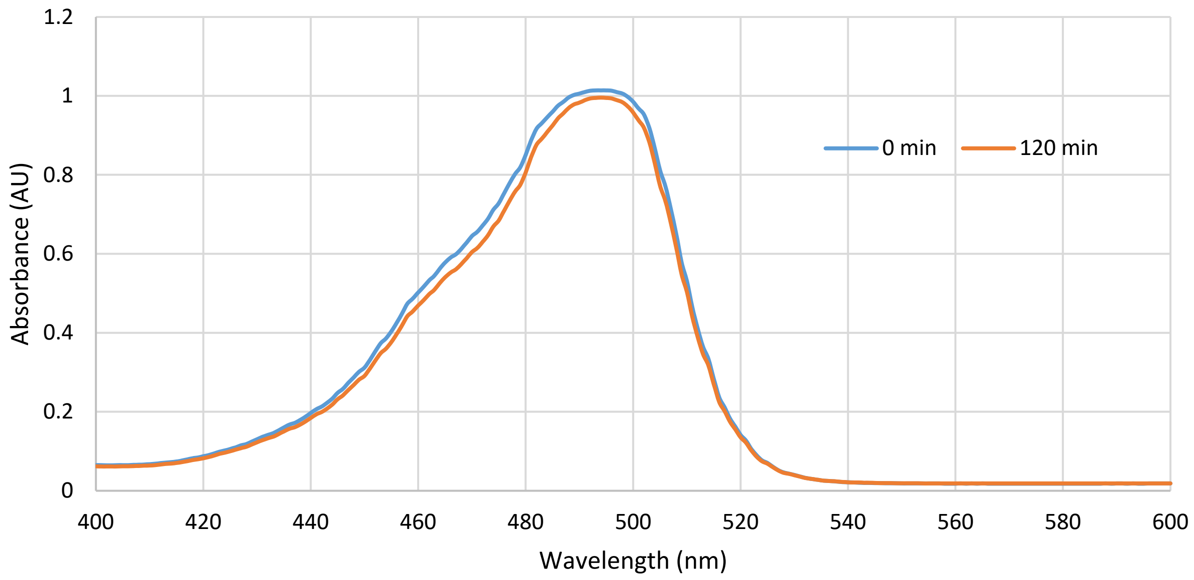

Figure S2. Absorbance spectra of RSSV-FITC (peak 495 nm) before and after UV exposure for 120 min.

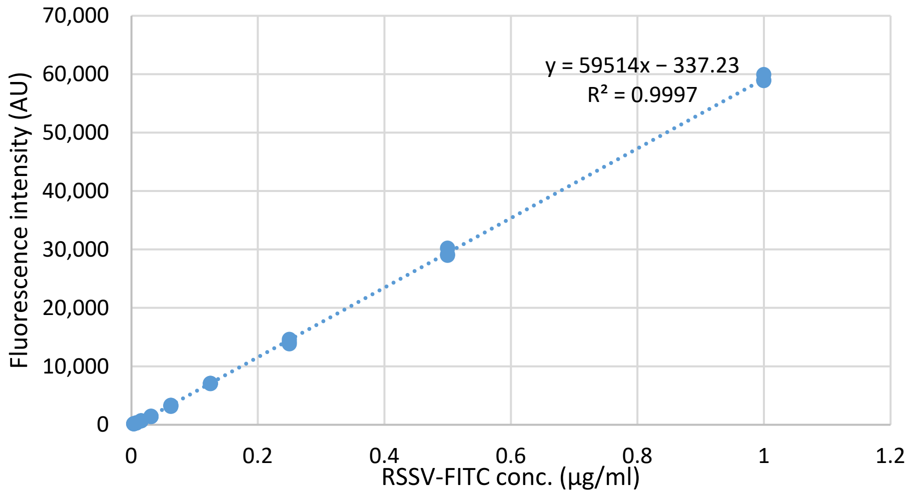

Figure S3. Calibration curve for the concentration of RSSV-FITC in solution.

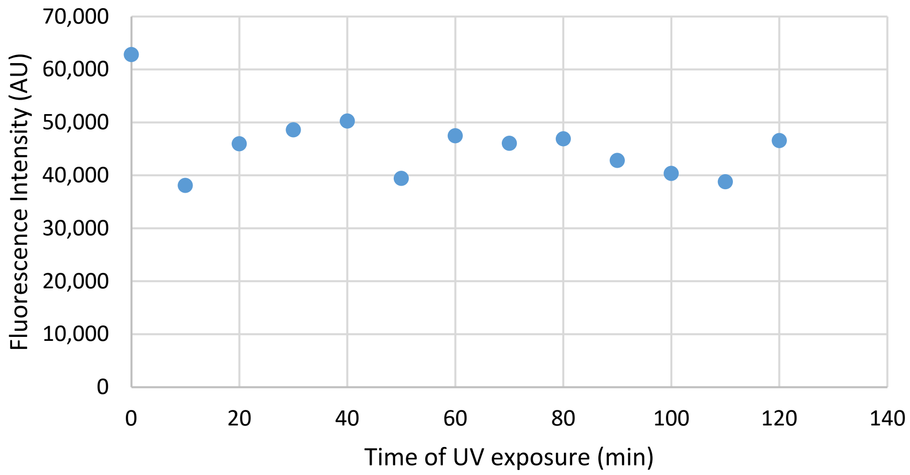

Figure S4. Fluorescence intensity decrease due to photo-bleaching for RSSV-FITC solution. There is a ~25% intensity decrease even after 5 min of UV treatment. After the initial drop in intensity, there is no further photo-bleaching.

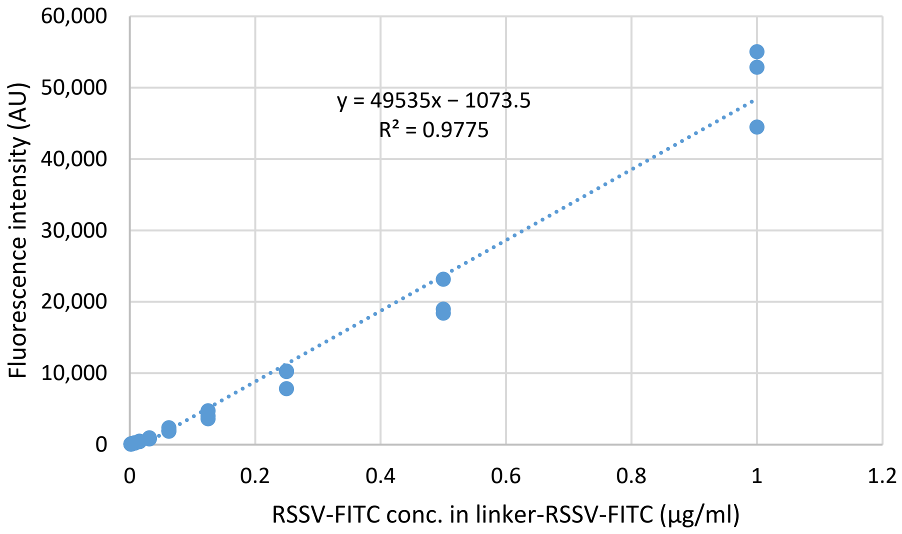

Figure S5. Calibration curve for fluorescence intensities of SNLD-RSSV-FITC solutions before UV treatment.

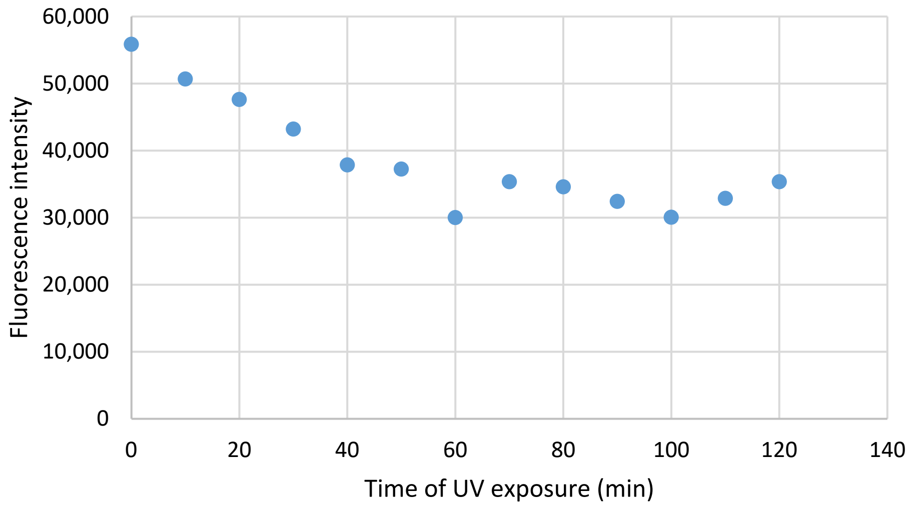

Figure S6. Fluorescence intensity decrease due to photo-bleaching for SNLD-RSSV-FITC solution. There is a ~35% intensity decrease gradually up to 60 min of UV treatment. After that drop in intensity, there is no further photo-bleaching.

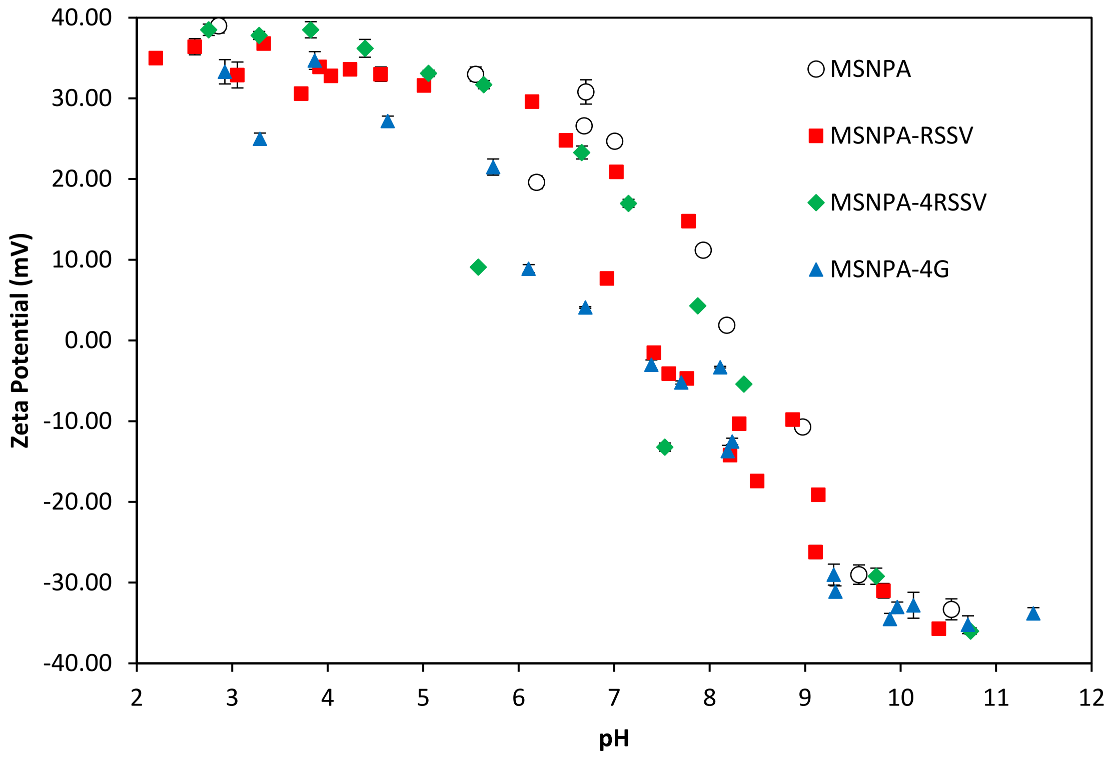

Figure S7. Comparison of zeta potential of MSNPA-RSSV, MSNPA-4RSSV and MSNPA-4G with bare MSNPAs measured over a wide range of pH.
